# Obstetric and perinatal outcomes in pregnant women with Takayasu’s arteritis: single centre experience over five years

**DOI:** 10.4274/jtgga.galenos.2019.2019.0115

**Published:** 2020-03-06

**Authors:** Liji Sarah David, Manisha Madhai Beck, Manish Kumar, Sudha Jasmine Rajan, Debashish Danda, Reeta Vijayaselvi

**Affiliations:** 1 Clinic of Obstetrics and Gynaecology, Christian Medical College and Hospital, Vellore, India; 2 Clinic of Neonatology, Christian Medical College and Hospital, Vellore, India; 3 Clinic of Obstetric Medicine, Christian Medical College and Hospital, Vellore, India; 4 Clinic of Rheumatology, Christian Medical College and Hospital, Vellore, India

**Keywords:** Takayasu’s arteritis, periconceptional counselling, vasculitis, chronic hypertension, high risk pregnancy

## Abstract

**Objective:**

To study obstetric and perinatal outcomes among pregnant women with Takayasu arteritis (TA), attending our hospital for pregnancy and childbirth between January 2011 to December 2016.

**Material and Methods:**

Retrospective study was carried out by abstracting clinical charts on all pregnant women with TA who underwent antenatal care and/or delivery in our hospital during this period. American College of Rheumatology criteria was used for diagnosis of TA. Sixteen women with TA were included in the study. Maternal demographic data, stage of disease, complications related to disease, details of treatment taken prior to pregnancy, pregnancy outcomes, and neonatal outcomes were studied.

**Results:**

Forty-four percentage (7/16) belonged to type 5 angiographic type, however the same proportion (7/16) had undergone surgical corrections prior to pregnancy and the majority (15/16) were on medical management. Only three women (19%) were diagnosed during pregnancy. Most did not have active disease measured by Kerr’s criteria (n=12; 75%), and Indıan Takayasu clinical activity scores A. Chronic hypertension was the commonest antenatal complication (56.2%), nearly one-third had growth restricted babies and 25% had preterm labour. There were no cardiovascular events, no maternal deaths, nor fetal or neonatal deaths. Two-thirds of our women were delivered by caesarean section.

**Conclusion:**

Preconceptional counselling is of paramount importance in women with TA. Good maternal and fetal outcomes are observed with close antenatal surveillance and multidisciplinary care. Pregnancy should be planned during disease remission, with good antenatal care, close monitoring of clinical symptoms, early diagnosis and treatment of complications.

## Introduction

Takayasu arteritis (TA), also called pulseless disease, is a chronic vasculitis that affects large vessels, mainly the aorta and its important branches (1). It was first described by Japanese ophthalmologist Mikito Takayasu in 1908 (2). The incidence of TA is 2,3 per million persons per year, with male to female ratio being 1:9 (3). Since young women are more commonly affected than men, one may encounter this condition in pregnancy, although it is still rare. Hence TA is also known as “young female arteritis” (2).

Management of pregnant women with TA is challenging because of the physiological increase in blood volume and cardiac output during pregnancy, which worsens the cardiovascular complications associated with the disease (4). Although the course of the disease per se is not affected by pregnancy, it has the potential to cause serious maternal and neonatal morbidity (5,6).

The inflammation of the aorta and its branches leads to stenosis, occlusion and aneurysm formation. Compliance of these blood vessels is also reduced leading to vasoconstriction, which in turn, leads to hypertension.

Pregnancy related increase in blood volume makes matters worse in the presence of “fixed” cardiac output, leading to increased cardiac strain. This often results in aortic regurgitation (AR) and congestive heart failure. Vasoconstriction also leads to chronic uteroplacental insufficiency, resulting in worsening of pre-existing hypertension superimposed pre eclampsia and/or fetal growth restriction (6,7).

There is a lack of recognized robust guidelines for management of such pregnancies and much of the evidence available in the medical literature is in the form of case series or case reports (6,7,8,9,10).

The aims of this study were to study the obstetric and perinatal outcomes among pregnant women with TA’s, attending our hospital for pregnancy and childbirth between January 2011 to December 2016.

## Material and Methods

This was a retrospective study carried out in the departments of Obstetrics, Neonatology and Rheumatology at Christian Medical College and Hospital, in Vellore, a tertiary care perinatal centre in India, which has an average of 15,000 deliveries per year. This study was approved by the institutional review board and ethics committee: IRB Min No. 10665 (Retro) dated 19.04.2017. The consent of pregnant women was not taken given the retrospective nature of the study.

Clinical charts of pregnant women with TA who underwent antenatal care and/or delivery in the Hospital between January 2011 to December 2016 were retrieved from the medical records department. American College of Rheumatology criteria were used for diagnosis of TA (Table 1).

Maternal demographic data, stage of disease based on angiographic classification, complications related to disease, C-reactive protein (CRP) and erythrocyte sedimentation rate (ESR) values, details of treatment taken (medical/surgical) prior to pregnancy, pregnancy outcomes, and neonatal outcomes were obtained.

Pregnancy outcomes studied were: existence of chronic hypertension and/or development of gestational hypertension; superimposed pre eclampsia; fetal growth restriction; any cardiovascular events in pregnancy; preterm labour; and the mode of delivery.

Neonatal outcomes included gestational age at birth, birth weight and any other neonatal complications.

**Figure 1:**
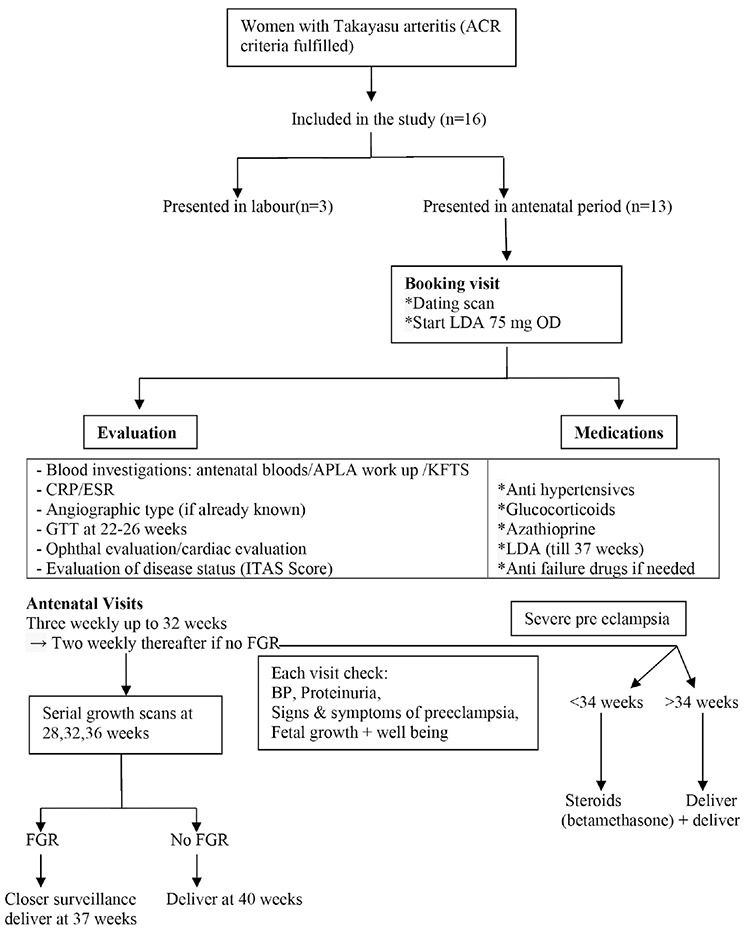
Flow diagram of clinical management in pregnant women with TA ACR: American College of Rheumatology, APLA: Anti-phospholipid antibody, KFTS: Kidney function tests, CRP: C-reactive protein, ESR: Erythrocyte sedimentation rate, LDA: Low dose aspirin, ITAS: Indian Takayasu arteritis society, FGR: Fetal growth restriction, GTT: Glucose tolerance test, FGR: Fetal growth restriction

Figure 1 shows the selection of patients for the study and clinical management of these women.

### Operational definitions

1. Active disease was defined as the presence of features of vascular ischemia or inflammation (such as carotodynia), claudication, diminished or absent pulse, bruit, asymmetric blood pressure in either upper or lower limbs or both, elevated ESR, systemic features, such as fever, musculoskeletal abnormality (without any other cause identified) and typical angiographic features.

New onset or worsening of two or more features indicated “active disease.” (Kerr’s Criteria 1994) (2).

2. Disease remission was defined as when there was complete resolution or stabilization of all clinical features and fixed vascular lesions (Kerr’s Criteria 1994) (2).

3. Angiographic types (11):

Type-1: Involves the branches from aortic arch.

Type-2 a): Involves the ascending aorta, aortic arch and its branches.

Type-2 b): Involves the ascending aorta, aorta arch and its branches and thoracic descending aorta.

Type-3: Involves thoracic descending aorta, abdominal aorta and or renal arteries.

Type-4: Involves abdominal aorta, and or renal arteries.

Type-5: Combined features of type 2B and 4.

4. Chronic hypertension: Blood pressure ≥140/90 that predates conception or presents before 20 weeks of gestation and persists >12 weeks postpartum (12).

5. Gestational hypertension: Blood pressure ≥140/90 in two occasions 15 minutes apart, identified for the first time after 20 weeks of gestation and normalizes by 12 weeks post-partum (12).

6. Preeclampsia: Presence of elevated blood pressure with protienuria (24hrs protein >300 mg, urinary protein creatine ratio >0.3, urine dipstick >1+) or creatinine >1.1, platelets <1,00,000, elevated liver enzymes twice the upper limit of normal range (12).

7. Superimposed preeclampsia: In women with chronic hypertension, new onset proteinuria after 20 week gestation, or a sudden inrease in proteinuria in those with pre-existing proteinuria, before 20 weeks or development of low platelets (12).

8. Secondary antiphospholipid antibody syndrome (APS): Antiphospholipid antibody, occurring secondary to an autoimmune disease (13).

9. Intrauterine growth restriction: Fetal weight less than the 10th centile for that gestational age (14).

10. Intra uterine death: Baby born after 28 weeks of gestation without any signs of life as defined by the World Health Organisation (15).

11. Indian Takayasu clinical activity scoring (ITAS): A clinical scoring system formulated by the Indian rheumatology association vasculitis group in March 2010, to study disease activity. Any new clinical manifestations of flare that has occurred over the previous three months are documented. A score ≥2 is considered disease activity (16).

12. ITAS.A scoring (ibid Oct 2012), ITAS including acute phase reactants (ESR, CRP). The score values of these are individually added to the original ITAS score, which gives an ITAS A-ESR or an ITAS A-CRP. A score of 5 or more is considered active disease.

The cutoff ranges for CRP and ESR were taken from ITAS.A. CRP <5 mg/dL had a score of 0, CRP of 6-10 mg/dL had score of 1, 11-20 mg/dL score of 2 and >20 mg/dL score of 3. Similarly ESR <20 mm/hr score of 0, 21-39 mm/hr score of 1, 40-59 mm/hr score of 2, and >60 mm/hr score of 3 (16). A higher score increases the likelihood of disease activity.

## Results

### Maternal characteristics

There were sixteen pregnant women with TA who were seen and delivered between January 2011 and December 2016. All women fulfilled the American College of Rheumatology 1990 criteria for diagnosis of TA. Three women were first diagnosed with TA during their pregnancies, or during intrapartum period when they presented in labour (Table 2). Remaining thirteen women who had been diagnosed with TA prior to pregnancy, were under regular follow up and treatment with rheumatologist, cardiologist or cardiothoracic surgeons, however they did not have any formal preconceptional counselling prior to becoming pregnant.

Nearly three quarters of the subjects did not have active disease, as per Kerr’s criteria, probably because they were under multidisciplinary care before pregnancy. However, the periconceptional disease status of 19% (3/16) women who either presented in advanced stage of pregnancy or were diagnosed to have the disease during pregnancy itself was unknown.

The majority (75%) were between 20-30 years of age (Table 2). The median (range) age of diagnosis was 23.5 (22.0-25.5) years. Median (range) diagnosis-to-pregnancy interval was 4.5 (2.25-8.00) years.

### Disease characteristics

The majority of subjects in whom the diagnosis of TA had been made prior to pregnancy (7/13; 54%), belonged to angiographic type 5 and none of them showed disease activity in pregnancy. We however did not know the angiographic type in three women who presented in advanced pregnancy without any pre-pregnancy evaluation having been performed earlier. As angiography is an invasive procedure, this was not performed during pregnancy solely for the purpose of diagnosis. The majority of women with hypertension had involvement of renal arteries too (6/9; 67%). Occular involvement was the next most common complication related to disease (see Table 3).

Nearly half the women in our cohort had undergone surgical intervention for TA prior to conception. Percutaneous transluminal angioplasty (PTA) with stenting of stenosed vessel was the most common surgical procedure carried out. Three women had stenting of the descending aorta, two of the common carotid artery, one underwent stenting of the subclavian artery and one had stenting of renal arteries along with the common carotid and subclavian artery. Two women required restenting following PTA due to restenosis of the arteries. One patient underwent nephrectomy for kidney failure; in this case renal function was 8%.

Almost all (15/16; 94%) were on medical therapy. These also included seven women who had also undergone PTA, with or without stent. All women were on multiple drugs, the most common combination (9/16; 56%), being steroids, immunosuppressants and anti-hypertensives. Seven women were on antiplatelet medications. One woman was on anti-cardiac failure drugs due to severe AR.

ITAS score was known for only half of cases, (n=8). The majority of them (n=7; 87.5%) had a score of <2 indicating lack of active disease, one had a score of 6. Using the ITAS.A criteria taking into account the values of acute phase reactants, ITAS.A-ESR/ITAS.A-CRP were calculated. The median (Inter Quartile range) for ITAS.A-ESR and ITAS.A-CRP were 2.50 (2.3) and 0.50 (0.2.75) respectively.

### Obstetrics and neonatal implications

Six (37.5%) women had miscarriages, prior to a successful pregnancy (Table 2). The reasons for miscarriages are not clear since they occurred elsewhere, without proper medical records. Nine women (56.2%) had chronic hypertension. Chronic hypertension was seen especially in the nine women in whom descending abdominal aorta and the renal arteries were involved (6/9; 66.7%). Two of these women developed superimposed preeclampsia (22.2%). None had secondary APS.

Cardiovascular events, such as congestive heart failure and worsening of AR were not encountered during pregnancy. One woman was on anti-cardiac failure medication prior to pregnancy due to severe AR, but did not have further deterioration of cardiac function during pregnancy. There were no maternal deaths in our cohort (Table 4).

In two women in our cohort no obstetric complications manifested. One was angiographic type 1, not on any treatment, and was in remission. The angiographic type of the other was unknown although she had bilateral ocular ischemic syndrome with dense cataract and was on steroids and aspirin.

Of the babies delivered nearly one third had fetal growth restriction and a quarter were born preterm. However, there were no intrauterine fetal deaths, either antepartum or intrapartum (Table 4).

Of the six women who delivered vaginally, two went into spontaneous labour whereas the rest had induced labour. Labour was induced in two women at term due to disease related complications of fetal growth restriction and superimposed preeclampsia whereas labour was induced for obstetric reasons, the preterm premature rupture of membranes, in the remaining two. Median (range) gestational age at delivery was 37 (36.00-38.75) weeks and median (range) birth weight was 2.6 (2.33-2.86) kg (Table 2).

Ten of the 16 (62%) deliveries were made by lower segment caeserian section (LSCS). Three of the LSCS were carried out as a result of disease condition per se: two had dilated aortic roots, at risk of aortic dissection during labour; and one had type 5 disease. The remainder of the LSCS (n=7) were done for obstetric indications, of which three for fetal distress, one for breech, one for arrest of descent, one for previous preterm LSCS with severe preeclampsia and one for previous LSCS not willing for Trial of labor (Table 4).

## Discussion

TA is a chronic idiopathic inflammatory disease affecting the aorta and large arteries. Etiology, though unknown, is probably autoimmune and is associated with diminished pulses, claudication, hypertension, stroke and cardiovascular complications. Histology shows panarteritis with acute exudative and chronic granulomatous inflammation associated with hyperplasia neovascularization. Aneurysms are caused by metalloproteases released from inflammatory cells, while infiltration by leukocytes and proliferation of myofibroblasts causes stenosis of vessels and its associated symptoms (17).

Since this condition is predominantly seen in young women, it is not uncommon to come across pregnant women with TA. In addition women with TA are more likely to become pregnant than other forms of vasculitis, since TA does not affect fertility (2,6,7). Mean age of diagnosis in our cohort was 24 years which was similar to other previous reports (18,19). Most of our patients were in the second decade of life as is classically described in the literature (2).

TA, being a Th1 mediated vasculitis, does well in pregnancy with successful outcomes (20). There have been many theories postulated for this such as an immunomoduatory effect of progesterones, release of cytokines by helper T cells and immunologic changes seen in pregnancy as part of an adaptive process (4,21,22). However it is associated with poor perinatal outcome, especially in patients with complicated disease and relapses (6,7,8,23).

Outcomes are affected by the type of arterial involvement. The incidence of hypertension, preeclampsia, and growth restriction in the fetus is found to be higher when renal artery and abdominal aorta are involved (24). Renin production is reported to increase when there is partial occlusion of the renal artery, which would explain the hypertension and decreased uteroplacental circulation resulting in growth restriction (24).

Relapses have also been found to be associated with preeclampsia and growth restriction, which could be the result of impaired placentation and fetal perfusion because of injury to the syncytiotrophoblast, endothelium of spiral veins, endovascular trophoblasts of the spiral arteries and glandular cells of the decidua by autoimmnune inflammatory processes (23). In addition the increase in blood volume and cardiac output during pregnancy can deteriorate the already existing vascular lesions of TA which could be fatal (4,21,24,25). The course of the disease however is not altered by pregnancy (20).

Pre-pregnancy counseling to stabilize the disease, is of paramount importance in women with TA (24,25). Presence of chronic hypertension, vasculitis and active disease six months prior to conception are factors associated with poor pregnancy outcomes (26). The main objective of periconceptional counselling is to assess disease activity; optimal control of blood pressure and changing over to safer drugs (20). There are various scores for assessing the disease activity such as Kerr’s score and ITAS score which will aid in counselling.

In our cohort although most of the women did not have periconceptional counselling, we had successful outcomes, secondary to good disease control prior to conception, and multidisciplinary management during pregnancy.

Suppression of the inflammatory process of TA and thus suppression of placental inflammation during pregnancy has been found to improve outcomes. Low dose corticosteroids and immunosuppressants are considered as the mainstay of treatment (23,27). Hidaka et al. (28) observed good pregnancy outcomes in their cohort on these medications. They had ten patients and nine of them were on steroids (28) One-fourth (n=4;25%) of our women were on corticosteroids alone and almost half (n=9:56%) on both steroids and immnunosupressants.Overall an 81% (13/16) of them were on steroids.

In our cohort, the preconceptional disease activity status was not known in 19% (3/16) cases as these presented in advanced pregnancy or in labour. This could be partially attributed to lack of patient awareness about the need for preconceptional counselling as well as the prolonged diagnosis to pregnancy interval. Unfortunately, this is not an uncommon scenario in the developing world where women do not have easy access to specialized health care .These women were diagnosed in the last trimester of pregnancy, one after 32 weeks when she first presented to our hospital, other two at 40 weeks when they presented in labour, and was incidently found to have diminished pulse in one limb. Singh et al. (18) showed that early assessment of disease status prior to conception and effective intervention prior to embarking on pregnancy, resulted in successful pregnancy outcomes. Other studies have noted increased rates of abortions in these women (24,25).

Though elevation of acute phase reactants may not be a very reliable marker for disease activation (29), it is considered a mode of assessing disease progression by ITAS.A (2012). This in turn would necessitate close antenatal surveillance with multidisciplinary involvement. Similar observations were included in other case reports and studies (2,8,10).

Almost half of our women were hypertensive and most of them required antihypertensive medication. Optimal control of hypertension is the key to successful pregnancy outcome since uncontrolled hypertension can cause: miscarriages; superimposed preeclampsia; abruption; fetal growth restriction; and ultimately intrauterine demise. Hypertension can also lead to catastrophic complications such as aortic dissections in women with dilated aortic roots. Thus hypertension, together with the autoimmune pathology of TA can directly or indirectly aggravate medical and/or obstetric complications (2,6,9,20,25,30).

Garikapati et al. (7) observed hypertension in 90% of their patients. In another similar study Singh et al. (18) found hypertension in 90% of patients with renal involvement. This however dropped to 50% following treatment for underlying pathology. Their rate of superimposed preeclampsia was only 10%. This is somewhat similar to our findings.

Although most of our women had type 5 TA, the severity of complications in terms of hypertension and superimposed preeclampsia was much lower than those mentioned by some others (7), since most of our women had already undergone treatment for the underlying disease prior to embarking upon pregnancy. Four of our patients with type 5 TA underwent PTA prior to pregnancy. The rates of hypertension reported by Kirshenbaum and Simchen (24) in his cohort who had antenatal counselling and stabilization of disease was 60%, which was very similar to our finding.

Pregnancy can also aggravate the already existing complication of TA, such as renal insufficiency, retinopathy, myocardial infarction, AR, aortic aneurysm, increasing maternal morbidity and mortality (2,20). One third of our cohort had renal involvement, 10% had cardiac and 15% had ophthalmic involvement. Although some of our women were diagnosed with AR prior to pregnancy, there was no deterioration in maternal cardiac function during pregnancy.

AR was the most significant cardiac problem as has been reported previously (7,18). In our cohort, two women had severe pre-pregnancy AR, with one being on anti-cardiac failure drugs. However, none of them had any worsening of symptoms during pregnancy. Aortic aneurysm and aortic dissection is a known complication of TA, and its occurrence in pregnancy with uncontrolled hypertension could cause maternal death, especially in the third trimester and during labour. We did not have any severe adverse outcomes such as any mortalities in our cohort. Close antenatal, intrapartum and postpartum monitoring, with a strict control of hypertension, and immediate surgical intervention whenever required, could prevent maternal deaths, as was observed by Lakhi and Jones (6) and Shafi et al. (8) in their case reports.

Delay in diagnosis, hypertension early in pregnancy and degree of vascular involvement (type 3, 4 and 5 TA) are considered predictors of poor outcomes in pregnant women with TA (4,9,10,30). We also noted similar findings. Women with type 5 disease had the most obstetric complications in terms of superimposed preeclampsia, preterm labour, fetal growth restriction and need for caesarean delivery.

The severity of the angiographic type determines the obstetric and perinatal outcomes, with more severe ones having a greater degree of adverse outcomes (7). This was also consistent with our findings (Table 5).

In our cohort, 31% babies were found to be growth restricted. The most common cause for fetal growth restriction was the presence of maternal hypertension, leading to uteroplacental insufficiency. Similar findings were noted previously by other authors (6,7,8,10,18,25). Women who had stenting and surgical corrections prior to pregnancy had well grown babies.

Involvement of infra-diaphragmatic arteries, especially the renal arteries is associated with adverse pregnancy outcomes (18,24,26). In our study, only one third women with involvement of the renal arteries had fetal growth restriction since most of these women were in remission. Disease activity was controlled with a combination of drugs such as immunosuppressants, steroids and anti-hypertensives in order to achieve optimal control of blood pressure. Singh et al. (18) had also reported improved outcomes in women who had angioplasty of the renal artery before conception.

Although mean gestational age at delivery was 37 weeks, 25% of our cohort had preterm birth, which was due to preterm premature rupture of membranes. This is somewhat more than the rate reported in the literature which is in the range 6-16% (18). We are unable to explain the increased incidence of preterm premature rupture of membranes in our cohort. Around 38% (6/16) of women had a history of miscarriages in our cohort. This is much higher than that found by Hauenstein et al. (2) who reported 12% rate. We are not aware of the cause of miscarriages in prior pregnancies as most of these were managed elsewhere with few clinical details available. It is quite possible that these would have been related to greater disease activity (Table 2). There was no intrauterine fetal demise, a severe and unfortunate complication of TA in the third trimester, nor any neonatal deaths, unlike in other studies were lack of multidisciplinary approach and uncontrolled disease status led to these unfortunate events (2,18).

Vaginal birth at term is recommended for all patients with TA (2,6). Most of our patients experienced spontaneous labour, whereas 25% underwent induction of labour. We had 60% LSCS rate in our cohort, the majority of which were done for obstetric indications. The rate of LSCS was similar to some previous reports (24,25). Women were more likely to have LSCS in severe disease types (type 4, 5) (Table 5). This is because the more severe angiographic types are more likely to have obstetric complications. Intrapartum fluctuations of blood pressure and increased cardiac output can worsen the already existing maternal complications of TA (24,25,28). It can further deteriorate the already existing uteroplacental insufficiency, leading to fetal compromise.

### Strengths

We had collected data over a period of five years with 16 patients included in the study. Ascertainment of data was very good with very few lost data.

### Study Limitation

This was a retrospective study and the clinical information obtained from the charts depended on the recordings of various clinicians.

## Conclusion

Periconceptional counselling is ideal in women with TA, however it may not be feasible in the developing world. Though most of our cohort conceived without preconceptional counseling, good outcome was achieved because of close antenatal surveillance and multidisciplinary care of such pregnancies. It is advised to plan pregnancy during disease remission, with good antenatal care and close monitoring of clinical symptoms. Early diagnosis of complications and its treatment result in good maternal and fetal outcome.

## Figures and Tables

**Table 1:**
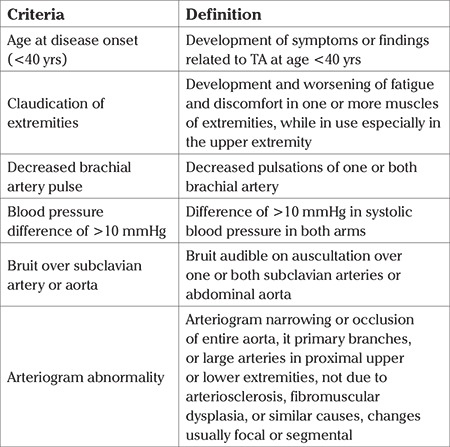
American College of Rheumatology 1990 criteria (31)

**Table 2:**
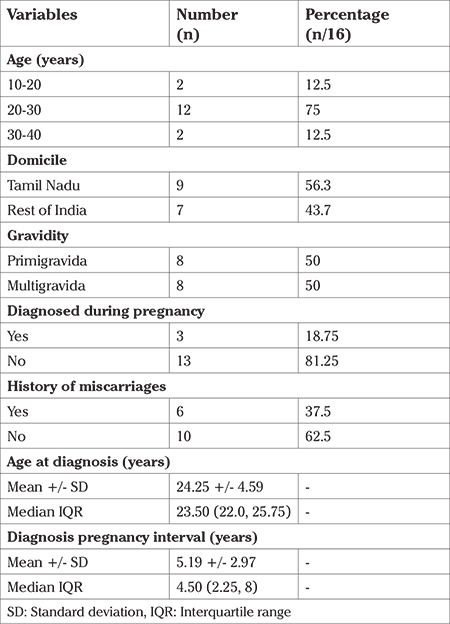
Maternal characteristics

**Table 3:**
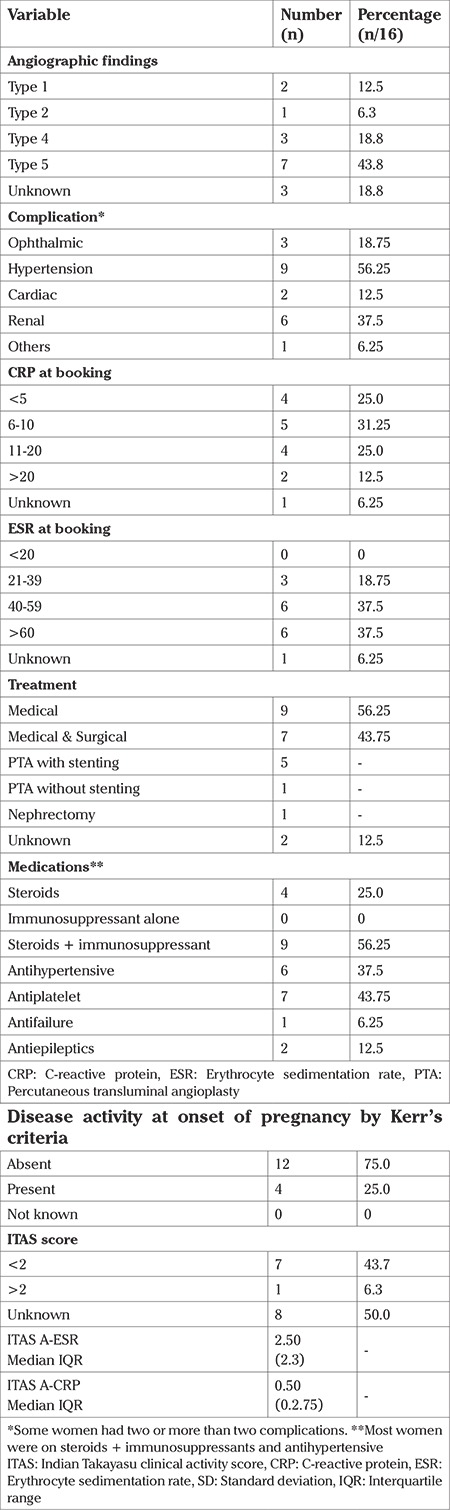
Disease characteristics

**Table 4:**
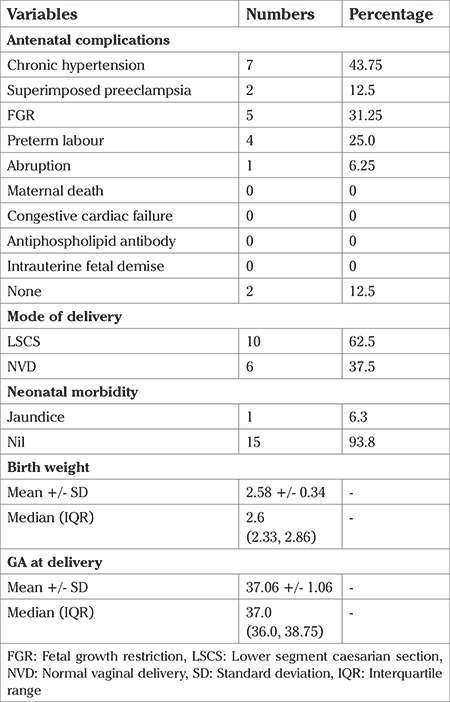
Maternal and neonatal outcomes

**Table 5:**
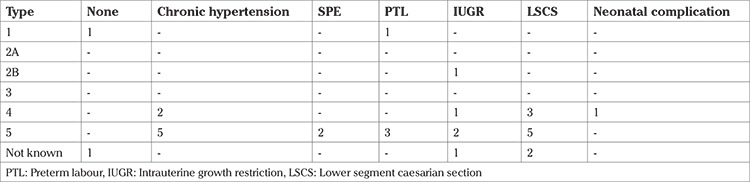
Obstetric and neonatal outcomes in different angiographic classification over period of January 2011 to December 2016
